# Supplementary material for: BTH Treatment Delays the Senescence of Postharvest Pitaya Fruit in Relation to Enhancing Antioxidant System and Phenylpropanoid Pathway
Source: Foods. 2021 Apr 13;10(4):846. doi: 10.3390/foods10040846 (PMC8069018; doi:10.3390/foods10040846)
Supplement: Supplementary file 1 [file foods-10-00846-s001.zip › Supplementary marterials/Figure S1-final.docx]

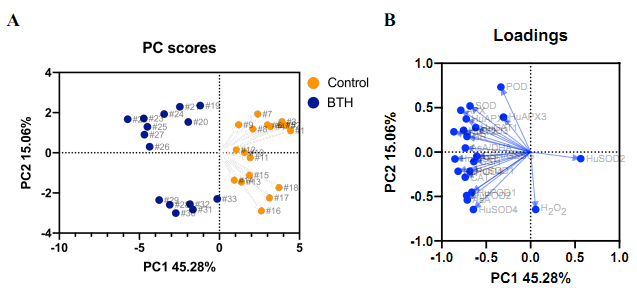


**Figure S1.** PCA scores analysis of the metabolites, enzymes and transcripts in antioxidant system in pitaya fruit after BTH treatment (A). Loadings plots for PCA of the metabolites, enzymes and transcripts in antioxidant system in pitaya fruit after BTH treatment (B). A total of 22 variables are considered, including the activities of SOD, CAT, APX, GR, POD, the content of H2O2, AsA and GSH, the ratio of AsA/DHA and GSH/GSSG, the mRNA transcripts levels of *HuSOD1*, *HuSOD2*, *HuSOD3*, *HuSOD4*, *HuCAT1*, *HuCAT2*, *HuAPX1*, *HuAPX2*, *HuAPX3*, *HuPOD1*, *HuPOD2* and *HuPOD4*. PC1 and PC2 account for 45.28% and 15.06% of the cumulative proportion of variance respectively.
