# Supplementary material for: BTH Treatment Delays the Senescence of Postharvest Pitaya Fruit in Relation to Enhancing Antioxidant System and Phenylpropanoid Pathway
Source: Foods. 2021 Apr 13;10(4):846. doi: 10.3390/foods10040846 (PMC8069018; doi:10.3390/foods10040846)
Supplement: Supplementary file 1 [file foods-10-00846-s001.zip › Supplementary marterials/Table S1.docx]

**Table S1.** Primers for Real-time PCR

| **Gene** | **Primer sequence (5’-3’) Forward** | **Primer sequence (5’-3’) Forward** |
| --- | --- | --- |
| *HuSOD1* | GCATGGCGAACCTCATCCTCTG | CTGGAACTGTCTCTGGTCTCAAGC |
| *HuSOD2* | ACCAGCATTGCCTGTGGTAA | GAGTCGCTGAGGCTGCTATT |
| *HuSOD3* | CTGCTCCAGAGCCTTGTTGA | GGGTTACTCCAATCCCGGTC |
| *HuSOD4* | CACATCCATGCCCTTGGTGA | ATGTTACCCAGATCACCCGC |
| *HuAPX1* | AGCTGCTCTTCGACACACAAGC | TCGCCTCCTACCTCCTCTCTCAG |
| *HuAPX2* | CTTCCGCGAATCCAAGCTCT | GCTGCCATCAGACAAAGCAC |
| *HuAPX3* | CAACCAATCGGCGGAAGATG | CAGGGTTTGAGGGACCTTGG |
| *HuCAT1* | ACGAGGGTCACAGAGAGCATCC | AACAACTTCAAGCAGCCTGGAGA |
| *HuCAT2* | CATGGAAGGGTTCGGTGTCA | CCCTGCGGCAATAGACTCAT |
| *HuPOD1* | TCTTCCACGACTGCTTCGTC | CGAGAATGTCGGAGCAGGAA |
| *HuPOD2* | TCTTCCACGACTGCTTCGTC | CGAGAATGTCGGAGCAGGAA |
| *HuPOD4* | AACCAGCTAGAGGTGCTGTTAC | CCCATAATGGCAAGCAGTCC |
| *HuACTIN* | TCTGCTGAGCGAGAAAT | AGCCACCACTAAGAACAAT |
